# Assessing the Impact of High Photon Energy Wavelengths on the Treatment of Chronic Neck and Shoulder Pain

**DOI:** 10.1155/2023/6672019

**Published:** 2023-10-04

**Authors:** Travis Sammons, Kirk Gair, Robert G. Silverman, Steve Shanks

**Affiliations:** ^1^Erchonia Corporation, Melbourne, FL, USA; ^2^Clinical Study Site, Laser Chiropractic, West Covina, CA, USA; ^3^Clinical Study Site, New York ChiroCare, White Plains, NY, USA

## Abstract

The effect of low-level laser therapy with high photon energy wavelengths, green and violet, for treating chronic musculoskeletal pain was examined in the first-ever clinical trial of its kind. Participants (*n* = 43) underwent a single 13-minute laser session. The primary measure of effectiveness was the change in initial visual analog pain (VAS) scores observed three minutes posttreatment. The success of a participant was defined in advance as a reduction of ≥30% in VAS scores, while the success of the study was predetermined as achieving a 65 ± 5% success rate among individual participants. Results demonstrated subjects' VAS pain scores decreased from 71.79 to 34.02 (*p* < 0.0001), while most participants in the study (81.4%) achieved a ≥30% decrease in pain scores. The findings from this clinical investigation provided substantial support for the first Food and Drug Administration clearance (K221987) for the combined application of green and violet lasers.

## 1. Introduction

Extensive documentation supports the effectiveness of low-level laser therapy (LLLT) in reducing pain and inflammation associated with various musculoskeletal disorders [1]; nonetheless, there remains limited investigation of high photon energy wavelengths, such as green light (495–570 nm) and violet/blue light (400–495 nm), for pain-related musculoskeletal ailments. Earlier research involving green and violet/blue wavelengths has largely focused on acne [2, 3], cellulite [4], fungi [5], and viruses [6, 7]. This clinical trial stands as the first known study to examine the specific effects of combining green and violet lasers on chronic musculoskeletal pain. Its findings mark a significant milestone in the field of research related to pain management.

Chronic neck and shoulder pain is widely recognized as one of the most common musculoskeletal disorders [8]. Neck pain is considered the fourth leading cause of disability, with a prevalence rate exceeding thirty percent on an annual basis [9]. While certain instances of neck pain may resolve spontaneously or with minimal treatment, studies show that nearly fifty percent of individuals will continue to experience varying degrees of pain or frequent recurrences.

The Food and Drug Administration (FDA) granted low-level laser market clearance for the reduction of neck and shoulder pain in 2002 for red laser diode alone [10] and in 2018 for the combination of red and violet laser diodes [11]. A previous comparative study analysed the difference between a red laser diode alone and a red/violet laser diode combination [12]. The primary objective of this nonrandomized noninferiority design trial was to determine whether the combination of violet and green lasers is as effective as the previously FDA-cleared treatments using either the red diode alone or the combination of red and violet laser diodes.

## 2. Materials and Methods

### 2.1. Study Subjects

The study recruited participants aged 18 years and older from two sources: patients seeking treatment for neck and shoulder pain within the investigators' patient pool and individuals who responded to local recruitment flyers and print ads. Participants did not receive any compensation for their involvement. To be eligible for the study, subjects had to exhibit symptoms of chronic neck or shoulder pain caused by osteoarthritic degenerative joint disorder, chronic muscle spasms, and cervical and thoracic spine sprain strain, as evidenced by their medication use history, medical records (including X-ray, MRI, and CAT scan reports), and physical examination. Chronic symptoms were defined as persisting for at least 30 days. Participants were required to agree to abstain from using over-the-counter or prescription medications, as well as herbal supplements intended for pain or inflammation relief, including muscle relaxants, throughout the study duration. They also had to refrain from other therapies for neck or shoulder pain, such as physical therapy, occupational therapy, hot or cold packs, chiropractic care, and acupuncture. Individuals were excluded from participation if they experienced primary pain outside or in addition to the neck or shoulder region, if the cause of their neck or shoulder pain could not be definitively diagnosed or if it was not attributed to osteoarthritis, chronic muscle spasms, or cervical and thoracic spine sprain strain, or if other potential contributing factors could not be adequately ruled out. Additional exclusion criteria included acute pain symptoms; active chronic pain diseases such as chronic fatigue syndrome or fibromyalgia; recent use of analgesics or muscle relaxants within seven days prior to the study treatment; systemic corticosteroid use (excluding inhaled and topical products); recent use of narcotics or botulinum toxin injection in the neck or shoulder within 30 days prior to the study treatment; history of cancer or cancer treatment within the past six months; unstable cardiac diseases (e.g., cardiac arrhythmia, congestive heart failure, and myocardial infarction); prior neck or shoulder surgery or herniated disc injury; presence of active infection, wound, or trauma in the planned treatment area; any medical or physical contraindications to light therapy; serious mental health illnesses such as dementia, schizophrenia, or psychiatric hospitalization in the last two years; pregnancy, breastfeeding, or planned pregnancy; and participation in another research study within the past 30 days.

### 2.2. Study Device

In this study, a portable nonthermal laser device was utilized, known as Erchonia® Model GVL. This handheld device employed a 520 nm semiconductor diode to emit visible green light and a 405 nm semiconductor diode to emit visible violet light. The device maintained a wavelength tolerance of ±10 nm. The laser diodes were designed with patented optics to generate a power output of 7.5 mW for the green laser and less than 5 mW for the violet diode. The procedure employed a predetermined pulse frequency in hertz (Hz); considering the 50% duty cycle generated by the pulse frequency, the total energy delivery was 4.88 Joules. To ensure participant safety, protective safety glasses were provided and required to be worn during all treatment procedures.

### 2.3. Study Procedure

Participants were administered a single 13-minute treatment using a nonthermal laser device on the day they enrolled in the study. The treatment protocol followed the same approach as previous studies, which utilized either a combination of red 635 nm and 405 nm violet diodes (Erchonia EVRL) [12] or a single red 635 nm diode (Erchonia PL2000) [13].

### 2.4. Study Endpoints

The baseline assessment for each participant took place on the day they enrolled in the study. This assessment included gathering demographic information, recording neck and shoulder VAS (visual analog scale) resting pain scores, and measuring the range of motion (ROM). The evaluation was repeated at 3, 24, and 48 hours after the administration of the laser treatment. The primary efficacy measure was the change in the neck and shoulder's VAS resting pain scores from baseline observed three minutes after the treatment. Individual subject success was defined as a reduction of at least 30% in the VAS scores. The overall success criterion for the study was predetermined as achieving 75 ± 5% of individual subject success in the current trial. The study was designed as a noninferiority study, comparing the effectiveness of the green/violet dual-diode laser to the red/violet dual-diode laser based on comparative data from a previous trial conducted in 2019.

Subject satisfaction was evaluated using a 5-point Likert scale, ranging from “very satisfied” to “not at all satisfied,” in response to the question: “Overall, how satisfied or dissatisfied are you with any change in the pain in your neck and/or shoulder following the study procedure with the study laser device?” This assessment was conducted at 24 and 48 hours.

### 2.5. Ethics

The study protocol received approval from a commercial institutional review board, WCG IRB Connexus® located in Puyallup, WA, under the study numbers 1290312 (Gair), 1291747 (Silverman), and 129557 (Comey). Before engaging in any study-related activities, all participants provided their informed consent by signing the necessary documentation.

## 3. Results

### 3.1. Subject Demographics and Clinical Characteristics

A total of 43 participants were enrolled in the study, consisting of 19 males (44%) and 24 females (56%). The average age of the participants was 48.32 years (standard deviation of 12.20 years), ranging from 21 to 82 years. 27 participants identified as Caucasian (63%), 11 as Hispanic (26%), 2 as African American (5%), 1 as Asian (2%), 1 as Caucasian or Asian (2%), and 1 as other (2%). The reported pain locations were as follows: right side (81%, *n* = 35), left side (84%, *n* = 36), back (91%, *n* = 39), right shoulder (70%, *n* = 30), and left shoulder (67%, *n* = 29). All reported pain locations were attributed to musculoskeletal causes. The average duration of neck and shoulder pain was 89.19 months (a standard deviation of 107.06 months), ranging from 1.0 to 362 months. The duration of over-the-counter (OTC) and/or prescription analgesic use for relieving neck and shoulder pain was as follows: 4 participants (22%) reported using them for less than 1 year, 4 (22%) for 1-2 years, 2 (12%) for 3–5 years, and 8 (44%) for more than 6 years. At the beginning of the study, the mean VAS pain rating on the 100-point visual analog pain scale (VAS) was 71.79.

### 3.2. Efficacy

The primary measure of efficacy, the mean VAS neck and shoulder pain score, exhibited a significant decrease from 71.79 to 34.02. A *t*-test for correlated samples confirmed a highly significant mean decrease of 37.77 points (*P* < 0.0001). A majority of the study participants (81.4%) achieved a ≥30% reduction in VAS scores, surpassing the upper limit of the overall study success criteria (70%–80%) by 1.4% and exceeding the lower limit by 11.4%. The progressive decrease in mean VAS scores continued from baseline to the study endpoint at 24 and 48 hours posttreatment, without further administration of the GVL laser. The total decrease in mean VAS scores from baseline to 48 hours posttreatment was 46.81 points, compared to 34.02 at the study endpoint immediately after treatment, as demonstrated in Table 1. In addition, the secondary measure of efficacy, neck and shoulder ROM, demonstrated improvement. The mean seated passive abduction increased by 33.14° on the right side and 31.28° on the left side. The mean shoulder measurements in the relaxed position improved by 32.67° on the right side and 31.16° on the left side, while the mean neck ROM measurements improved by 17.18° on the right side and 16.66° on the left side. At the study endpoint, participant satisfaction with the study outcomes was predominantly rated as “very satisfied” (72%, *n* = 31). A smaller proportion indicated being “somewhat satisfied” (26%, *n* = 11), and only one participant (2%) selected “neither satisfied nor dissatisfied.” None of the participants rated their satisfaction as “not very satisfied” or “not at all satisfied.” The high level of satisfaction, with the majority reporting being “very satisfied,” persisted throughout the study evaluation, including the 48-hour postprocedure evaluation. A comparison of clinical outcomes of the green/violet laser compared to the FDA-cleared violet/red and red laser alone studies is demonstrated in Table 2.

### 3.3. Safety

No adverse events were reported by any subject throughout the study duration.

## 4. Discussion

In this study, the short wavelengths utilized were 520 nm and 405 nm, with photon energies of 2.4 eV and 3.0 eV, respectively. These observed photon energies are notably higher than those commonly employed in low-level laser therapy (LLLT); in fact, the violet 405 nm wavelength (3.0 eV) has twice the energy of an infrared wavelength of 808 nm (1.5 eV). The data from this clinical trial provide imperative evidence that short wavelengths in the visible spectrum are effective for deep-seated musculoskeletal pain. The therapeutic effects of deep tissue provided by green and violet wavelengths are supported by photochemistry, which is a process involving the absorption of light and not direct penetration. According to the first law of photochemistry, in order for low-power visible light to produce any impact on a living biological system, photons must be absorbed by specific electronic absorption bands associated with molecular chromophores or photo acceptors [14]. Visible light absorption by the mitochondrial electron transport chain (ETC) is a crucial process. These organelles are central to generating cellular energy through oxidative phosphorylation. The ETC consists of four protein complexes—complex I (NADH-Q oxidoreductase), complex II (succinate-Q reductase), complex III (cytochrome C reductase), and complex IV (cytochrome c oxidase). Proton gradients are created within the ETC, enabling the synthesis of adenosine triphosphate (ATP), the primary energy currency of cells.

**Figure 1:**
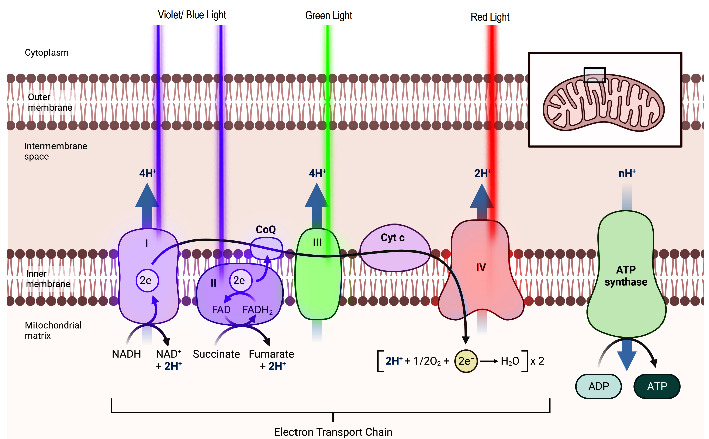
Absorbance of light in the mitochondrial electron transport chain.

The red wavelength range (620–750 nm) has been the subject of extensive research, particularly due to its interactions with complex IV (cytochrome c oxidase) [15]. As the terminal enzyme in the respiratory chain, complex IV plays a vital role in facilitating the transfer of electrons from cytochrome c to molecular oxygen. While complex IV absorption bands coexist at the blue/violet and green wavelengths [15], there is evidence that other primary ETC complexes are responsible for the absorption of short wavelengths. Complexes I and II are acceptors of high-energy electrons from the Krebs cycle and have a peak absorption band in the violet/blue spectra [16–18]. These two complexes are responsible for shuttling high-energy electrons (NADH and FADH2) to ubiquinone followed by complex III. Throughout their progression within the chain, electrons undergo a transition from higher energy levels to lower energy levels. Consequently, complex III has a peak absorption band in the green wavelength spectra [18–20], which is lower than the violet/blue wavelengths, as demonstrated in Figure 1.

Following the absorption of violet/blue and green wavelengths, complexes I, II, and III experienced heightened activity and augmented proton gradients. This, in turn, leads to an increase in ATP production. Notably, complexes I and III are highly susceptible to loss of activity and impairment due to aging [21], chronic conditions [22, 23], and pharmacological drugs [24–26], leading to the loss of oxidative phosphorylation. In individuals with osteoarthritis (OA), it has been observed that dysfunction in chondrocytes and mitochondrial electron transport chain (ETC) activity resulted in reduced activity of complexes I, II, and III when compared to chondrocytes in a normal state [27]. Dysfunction in complexes I, II, and III can have a significant impact on multiple pathways linked to cartilage degradation. These pathways encompass oxidative stress, impaired chondrocyte biosynthesis and growth responses, heightened inflammation and matrix catabolism induced by cytokines, cartilage matrix calcification, and elevated chondrocyte apoptosis. Other photoacceptors in human tissue include flavoproteins, which are key enzymes in the early stage of mitochondrial respiration, have absorption in the violet spectrum [28]. Flavoproteins participate in a wide range of biological processes, including the generation of ATP, bioluminescence, neutralization of oxidative stress-induced radicals, DNA repair, and modulation of apoptosis. Porphyrins, renowned for their remarkable photosensitizing capabilities that facilitate the production of reactive oxygen species by transferring energy to oxygen atoms in their ground state, exhibit significant absorption bands within the green spectrum at wavelengths of 502 nm, 540 nm, and 560 nm [29] and violet at approximately 400 nm [30]. At lower levels of radiation exposure, the presence of porphyrin facilitates the generation of singlet oxygen through energy transfer. This singlet oxygen, in turn, activates the redox activity within the respiratory chain, enhancing chemiosmosis and triggering an influx of calcium ions, thereby promoting mitosis. The unique photoacceptors of green and violet/blue light create a wide array of photobiological effects. The following investigational abstracts exhibit histological evidence subsequent absorption of green and violet/blue light.

### 4.1. Histological Evidence

In a study conducted by Fukuzaki et al., it was shown that transcranial stimulation using a green low-power laser resulted in increased levels of protein kinase B (Akt) after a 4-hour period, as compared to the nonirradiated control group [31]. Akt assumes a pivotal role in maintaining the delicate balance between cellular survival and programmed cell death pathways. A growing body of evidence supports the involvement of Akt in various cellular processes, including glucose metabolism, transcriptional regulation, apoptosis, cell proliferation, migration, and angiogenesis.

Merigo et al. conducted a study revealing the beneficial impact of green lasers on the process of osteogenic differentiation in murine bone marrow stromal cells (BMSCs) [32]. Bone marrow stromal cells (BMSCs) play a crucial role in bone repair as they release factors that promote natural healing mechanisms and can differentiate into osteoblast-like cells to contribute to new bone formation. In addition, BMSCs have the potential to support nerve regeneration [33].

Anwer et al. conducted a study to examine the stimulatory impact of a green laser on the proliferation and mitochondrial activity of adipose-derived stem cells (ADSCs) [34]. The results indicated that green laser light led to a significant increase in proliferation, which was attributed to increased mitochondrial activity. The authors observed that NADH and flavins, essential components of the mitochondrial respiratory chain, exhibited stimulation at a wavelength of 532 nm. These molecules actively participate in the redox reactions occurring within the inner mitochondrial membrane. The redox reaction leads to an elevation in ATPase activity, which impacts the flux of calcium ions (Ca^2+^) influencing the levels of cyclic nucleotides, thereby regulating the processes of DNA and RNA synthesis.

According to Kassák et al., exposure to a low-power green laser resulted in a notable thirteen percent rise in the mitochondrial transmembrane potential [35], which is integral to cell life, and normal cell function is essential for ATP synthesis.

The effects of red, green, and infrared light sources on the peripheral nervous system were investigated by Lubart et al. [29]. No significant difference was found for the action potential of the nerve between the infrared and control group, which means infrared light affects the nerve minimally or not at all. The findings revealed that only the red and green wavelengths had an impact on the compound action potential of the nerve, with green light demonstrating significantly greater effectiveness than red light. The authors concluded that visible light (630 nm and 540 nm) is recommended for nerve regeneration.

O'Connor et al. conducted a study to explore the effects of a single treatment utilizing three distinct wavelengths (635 nm, 532 nm, and 405 nm) on the established hallmarks of renal fibrosis [36]. The findings indicated that the effects observed were specific to each wavelength. The 405 nm wavelength exhibited the most pronounced impact on reducing the number of apoptotic cells. Conversely, only the 532 nm green laser demonstrated a significant reduction in TGF-B content, a crucial factor implicated in fibrosis development.

Hwang et al. conducted a study to explore the anti-inflammatory properties of LLLT at different wavelengths (650 nm, 532 nm, and 405 nm) on annulus fibrosus cells in the context of intervertebral disc degeneration [37]. While all visible light wavelengths significantly decreased interleukin 8, only the 405 nm violet laser inhibited the secretion of interleukin 6. Research suggests that interleukin 6 (IL-6), a cytokine known for its proinflammatory properties, plays a significant role in the development of neuropathic, inflammatory, and neurological pain in disc tissues. Furthermore, IL-6 is also implicated in the pathogenesis of rheumatoid arthritis. Notably, 24 h after irradiation, the inhibition of interleukin 8 was measured at wavelengths of 650 and 532 nm, while at 405 nm had no significant change. However, 96 h after irradiation, only 405 nm showed a significant inhibitory effect. This phenomenon could explain the outcomes of the current neck and shoulder pain study utilizing Erchonia GVL, as a significant reduction in pain was demonstrated immediately following treatment and sustained for 48 hours posttreatment. The green laser could be the primary contributor to immediate proinflammatory inhibition, while the violet laser contributes to deferred proinflammatory inhibition at 48 hours posttreatment.

Pope et al. assessed the light-induced release of nitric oxide (NO) from cyclooxygenase (COX) utilizing different wavelengths, including 447 nm, 532 nm, 635 nm, and 808 nm [38]. The exposure to 447 nm blue light resulted in a substantial 33.6% increase in the release of nitric oxide (NO). A biphasic response pattern was observed for all wavelengths, indicating an optimal exposure range of 2.16–2.88 J/cm^2^ for maximal responsiveness. It was noted that the immediate elevation of cellular NO levels is highly influenced by the source of electrons entering the electron transport chain.

Serrage et al. reported that exposure to a 400 nm violet laser resulted in a significant 53% increase in maximal respiratory rates in myotubes [39], which are skeletal muscle fibers formed through myoblast fusion during the developmental stage.

Kushibiki et al. conducted a study to examine the impact of different wavelengths, specifically 405 nm, 664 nm, and 808 nm, on the production of reactive oxygen species (ROS) in various types of cells [40]. Following irradiation with the blue laser, intracellular levels of reactive oxygen species (ROS) showed a significant increase in all cell types. However, irradiation with red or near-infrared lasers did not induce intracellular ROS generation. ROS serve as important secondary messengers that influence cellular homeostasis. The mechanism behind ROS production involves a photosensitization process with flavins for light within the 400–500 nm range, while longer wavelengths may directly generate ROS from mitochondria.

The summary of histological evidence is demonstrated in Table 3.

Short wavelengths that deliver high-energy photons may produce unique photochemical reactions that are not observed at longer wavelengths. When a photoexcited electron drops, excess energy is emitted as a photon from the fluorescent light. The wavelength of the fluorescent photon will be “red-shifted” to a less blue wavelength than the originally absorbed photon. This fluorescent photon will trigger further photochemical actions by being absorbed into another molecule to set off another chain of electron transfer events [41]. Biophotons are an example of a photon released through fluorescence. Chromophores, such as porphyrin rings, flavin, pyridinic rings, lipid chromophores, and aromatic amino acids, have the capacity to capture biophotons. These biophotons can be absorbed by photosensitive biomolecules in cells and neurons, which then transfer the absorbed energy to nearby biomolecules through resonance energy transfer. This phenomenon triggers conformational changes and initiates complex signalling processes within and between cells. Furthermore, the systemic influence of LLLT photons occurs in triplets. While the abovementioned fluorescence photons result in immediate emission to another molecule, phosphorescence photons emitted from triplets are delayed emissions that persist for some time, even after laser treatment.

Along with the emission of high-energy photons, short wavelengths play an important role in the autonomic nervous system (ANS). While red visible light stimulates the sympathetic nervous system, middle frequencies such as green light balance physiology and high energy/short wavelengths activate the parasympathetic nervous system [42]. Autonomic nervous system (ANS) imbalance has been shown to play a direct role in musculoskeletal chronic pain. The withdrawal of parasympathetic activity can promote the dominance of sympathetic responses in the autonomic nervous system (ANS), leading to insufficient stress responses and cardiovascular adjustments during postexercise recovery or sleep. Increased sympathetic activity can activate pain-sensing nerve fibers, leading to widespread pain and heightened sensitivity to both painful (hyperalgesia) and nonpainful (allodynia) stimuli. Hallman et al. conducted a study involving 24-hour ambulatory monitoring of an electrocardiogram (ECG) in individuals with chronic neck and shoulder pain as well as in healthy control subjects [43]. The presence of pain in individuals was associated with an elevated heart rate and a decrease in heart rate variability observed during sleep, indicating an increase in sympathetic activity and a decrease in parasympathetic activity.

## 5. Conclusions

This novel study marked the first instance of showcasing the efficacy of high-energy photon wavelengths in alleviating chronic pain associated with the neck and shoulder areas. Eighty-one percent of the participants in this research met the predetermined success criteria of thirty percent or more reduction in pain immediately following a single treatment. Furthermore, participants reported a continued improvement in pain scores even forty-eight hours after the treatment. This discovery may pave the way for future studies with extended follow-up periods to assess the duration of the laser's analgesic effects. These findings strongly suggest that the combined use of green and violet lasers synergistically reduces pain in individuals dealing with chronic neck and shoulder discomfort, offering a safe and medication-free alternative treatment.

## Figures and Tables

**Table 1:** VAS ratings across study duration.

	(*n* = 43)	
	Mean	SD
Preprocedure	71.79	11.60
Study endpoint	34.02	20.99
24-hour postprocedure	29.12	21.61
48-hour postprocedure	24.98	23.00

**Table 2:** Clinical outcomes of the green/violet laser compared to the violet/red laser and red laser alone.

	Green and violet combination	Red and violet combination	Red laser alone
Subjects	43	44	43
Duration of pain (months)	89.19	76.58	61.7
Subjects meeting study success criteria, ≥30% pain reduction	81.4%	75%	65%
(%) improvement in VAS from baseline to immediately after treatment	52.61%	45.84%	48.17%
(%) improvement in VAS from baseline to 48 hrs posttreatment	65.20%	50.17%	43.19%

**Table 3:** Summary of histological evidence.

References	Green wavelength	Blue/violet wavelengths
Fukuzaki et al. [30]	+ Protein kinase B (PKB)	
Merigo et al. [32]	+ Bone marrow stromal cells (BMSCs)	
Anwer et al. [34]	+ Proliferation and mitochondrial activity of adipose-derived stem cells (ADSCs)	
Kassák et al. [35]	+ Mitochondrial transmembrane potential	
Lubart et al. [29]	**+** Compound action potential (CAP)	
O'Connor et al. [36]	− Transforming growth factor *β*	− Apoptotic cells
Hwang et al. [37]	− Proinflammatory cytokine interleukin 8	− Proinflammatory cytokine interleukins 6 and 8
Pope et al. [38]		+ Cellular nitric oxide (NO) levels
Serrage et al. [39]		+ Myotubes
Kushibiki et al. [40]		+ Biomodulation of reactive oxygen species (ROS) production

+ Upregulation; − downregulation.

## Data Availability

The data involved in this study are available from the corresponding author upon request, and privacy-related parts of the patient will not be provided.
